# Short- and intermediate-term impact of DTC telemedicine consultations on subsequent healthcare consumption

**DOI:** 10.1007/s10198-023-01572-z

**Published:** 2023-02-24

**Authors:** Cecilia Dahlgren, Emma Spånberg, Sofia Sveréus, Margareta Dackehag, Per Wändell, Clas Rehnberg

**Affiliations:** 1 Department of Learning, Informatics, Management and Ethics, Karolinska Institutet, Tomtebodavägen 18 A, LIME, 171 77 Stockholm, Sweden; 2 Region Stockholm, Center for Health Economics, Informatics and Healthcare Research, Stockholm, Sweden; 3 Region Dalarna, Department of Analysis, Falun, Sweden; 4 Department of Economics, Lund University, Lund, Sweden; 5 Department of Neurobiology, Care Sciences and Society, Division of Family Medicine and Primary Care, Karolinska Institutet, Stockholm, Sweden

**Keywords:** Telemedicine, e-health, Primary healthcare, Healthcare consumption, Interrupted time series analysis, Sweden, I110, I120, I180, O330

## Abstract

**Aim:**

The use of direct-to-consumer (DTC) telemedicine consultations in primary healthcare has increased rapidly, in Sweden and internationally. Such consultations may be a low-cost alternative to face-to-face visits, but there is limited evidence on their effects on overall healthcare consumption. The aim of this study was to assess the short- and intermediate-term impact of DTC telemedicine consultations on subsequent primary healthcare consumption, by comparing DTC telemedicine users to matched controls in a Swedish setting.

**Methods:**

We constructed a database with individual-level data on healthcare consumption, for all residents of Region Stockholm in 2018, by linking national and regional registries. The study population included all individuals who had ≥ 1 physician consultation (telemedicine or face-to-face) during the first half of 2018. DTC telemedicine users were matched 1:2 to controls who were non-users of DTC telemedicine but who had a traditional face-to-face consultation during the study period. The matching criteria were diagnosis and demographic and socioeconomic variables. An interrupted time series analysis was performed to compare the healthcare consumption of DTC telemedicine users to that of the control group.

**Results:**

DTC telemedicine users increased their healthcare consumption more than controls. The effect seemed to be mostly short term (within a month), but was also present at the intermediate term (2–6 months after the initial consultation). The results were robust across age and disease groups.

**Conclusion:**

The results indicate that DTC telemedicine consultations increase the total number of physician consultations in primary healthcare. From a policy perspective, it is therefore important to further investigate for which diagnoses and treatments DTC telemedicine is suitable so that its use can be encouraged when it is most cost-efficient and limited when it is not. Given the fundamentally different models for reimbursement, there are reasons to review and possibly harmonise the incentive structures for DTC telemedicine and traditional primary healthcare.

## Introduction

Telemedicine consultations in primary healthcare have increased substantially in recent years and this trend has been further accentuated by the COVID-19 pandemic. During a telemedicine consultation, healthcare personnel and patients are spatially separated and interact through video link, telephone, or chat. The consultations can either be integrated into the provision of care at primary healthcare centres or target patients directly, so-called direct-to-consumer (DTC) telemedicine. Providers of DTC telemedicine have no connection to the patients’ primary healthcare centres whereas primary healthcare providers offer both integrated telemedicine consultations and face-to-face consultations.

DTC telemedicine, which is the focus of this study, has been the subject of a heated debate on whether the benefits of the services outweigh the costs. DTC telemedicine consultations have the potential to lower costs on the provider side and decrease travel time and loss of production on the consumer side [1–4]. Moreover, they have benefits related to accessibility and a reduced risk of spreading disease. Shortage of physicians at primary healthcare centres limits access to face-to-face primary healthcare. The DTC telemedicine sector can cope with such shortage more flexibly using free capacity across geographical areas. However, a major concern is that the convenience of DTC telemedicine may create an increased demand for healthcare for less severe conditions, leading to overuse of healthcare services and increased expenditure. Another concern is that DTC telemedicine interactions are not sufficient for properly examining a patient. As a result many of these consultations may need to be followed up by a physical examination through a face-to-face consultation [5–7]. The question of how DTC telemedicine impacts on subsequent healthcare consumption has become highly policy relevant and requires investigation in order to shape the future role of DTC telemedicine in healthcare.

Since telemedicine is a relatively new phenomenon, rigorous analyses of its effects on subsequent healthcare consumption are scarce. Many studies target only specific patient groups [8–13]. The focus is mainly on the short-term impact within care episodes, without taking the level of consumption in the pre-intervention period into account [3, 14–17]. Furthermore, previous studies report varying results regarding how access to telemedicine affects overall healthcare consumption. Some studies indicate that telemedicine triggers additional face-to-face consultations or has a minor impact on the consumption [5, 18–21], whereas other studies suggest that telemedicine consultations have a potential to replace face-to-face consultations, at least partially [22–24].

The diverse findings from previous research suggest that the impact of telemedicine may depend on how, in what context, and for what types of conditions the consultations are offered. Most previous studies have been conducted in a US context and focus mainly on adult patients with private insurance. This makes it difficult to generalise research results to healthcare systems with universal coverage, like the Swedish system, where DTC telemedicine is largely tax funded and available to the entire population.

In Sweden, there was a rapid increase and broad adoption of DTC telemedicine consultations, even before the COVID-19 pandemic, which makes the country a relevant case for analysing how these consultations affect healthcare consumption. This study contributes to the growing body of literature focusing on the Swedish experience of DTC telemedicine by adding knowledge about the short-term (within a month) and intermediate-term (2–6 months after the initial consultation) effects of DTC telemedicine consultations on subsequent healthcare consumption.

### Aim

The aim of this study was to assess the short- and intermediate-term impact of DTC telemedicine consultations on subsequent primary healthcare consumption, by comparing users of DTC telemedicine to matched controls in a Swedish setting. We assessed the impact on the consumption of face-to-face and DTC telemedicine consultations, in combination and separately.

### Analytical framework

Based on previously published studies, we hypothesised that four mechanisms might affect subsequent healthcare consumption among users of DTC telemedicine consultations: 1) technology, 2) provider incentives, 3) case mix and 4) patient healthcare-seeking behaviour. We classified the first three mechanisms as having short-term impacts and the fourth mechanism, if present, as having an intermediate-term effect.

The telemedicine *technology* enables patients and healthcare providers to be spatially separated. However, for many types of health problems, a medical examination requires the presence of the patient. Consequently, in some cases, telemedicine consultations may not be sufficient for solving the health issue and may, therefore, lead to additional consultations to a greater extent than face-to-face consultations. The effect is likely to be short term, as the additional consultation will take place within the same care episode. Previous studies have found mixed results regarding the diagnostic accuracy of telemedicine versus face-to-face consultations. Shi et al. [10] found that telemedicine providers ordered less appropriate testing for acute respiratory infection diagnoses, which indicates less diagnostic accuracy. In contrast, Hertzog et al. [14] did not observe any differences in diagnostic accuracy for low-acuity illnesses between telemedicine and face-to-face consultations.

In addition to the potential need for a face-to-face medical examination, Bavafa et al. [18] argued that telemedicine consultations might not provide enough information exchange between patients and providers, which could increase the demand for additional consumption. Similarly, Farr et al. [6] argued that the communication between patient and physician during a DTC telemedicine consultation may reduce the ability to discuss treatment decisions and to use shared decision-making. This may result in patients feeling a need for further information and an additional consultation.

Hence, even if there are ambiguous findings, the inherent limitation of the technology may, at least in relation to some health problems, increase the likelihood for additional subsequent face-to-face consultations in the short term.

It is well known that the design of reimbursement systems gives different *incentives* that impact on provider behaviour in terms of accessibility and level of service provision [25]. Reimbursement models in Swedish primary healthcare range from payment per contact to various degrees of capitation payment combined with fee-for-service. In Region Stockholm, capitation is the dominant model and accounts for 60 percent of the total compensation to traditional primary healthcare centres [26]. The DTC telemedicine sector, on the other hand, is dominated by independent private providers reimbursed through payment per contact models [27]. With the capitation principle, the financial incentives for additional consultations are weak. Therefore, the capitation principle may limit access and could lead to delayed treatment and greater resource requirements. In contrast, telemedicine providers with reimbursement by payment per contact have no financial incentives to prevent overuse, which could result in consultations that would not have taken place in the traditional primary healthcare setting. Consequently, there is a risk of more unnecessary follow-up consultations with a payment per contact model.

Differences in *case mix* between users of DTC telemedicine and users of face-to-face consultations is another factor that could impact on subsequent utilisation. Recent research on patient characteristics of users of DTC telemedicine compared with face-to-face consultations found significant differences in patient characteristics between the users of the two types of healthcare services [27]. In addition, the diagnosis range and conditions might be less severe among DTC telemedicine users, due to the lower thresholds for accessing DTC telemedicine. Users of DTC telemedicine may have fewer subsequent consultations than face-to-face patients in the short term because their conditions are less severe. Although we matched DTC telemedicine users to controls based on diagnosis, among other characteristics, we could not eliminate a potential impact of a remaining, systematic but unobservable variation in health within each visit diagnosis.

A fourth mechanism refers to how the use of DTC telemedicine may influence a patient’s *healthcare-seeking behaviour* in the intermediate term. After an initial encounter with DTC telemedicine, installation of the app, and learning how it works, the barrier to reusing the services may be lower. This effect is likely not immediate, but will manifest the next time the individual needs healthcare services. Previous research suggests that patients experiencing lower barriers to primary care have a higher propensity to seek healthcare, especially for minor complaints [28]. A high number of DTC consultations in the intermediate term could indicate a previously unmet demand for healthcare that is being satisfied or that the new technology has made treatment more accessible and convenient for some patients [5]. On the one hand, this may lead to earlier detection and treatment, which can decrease the risk of illness progression. On the other hand, low access barriers may lead to unnecessary or low-value treatment [28].

The distinction between short-term and intermediate-term effects in the study is justified by the fact that the possible mechanisms mentioned above arise at different times. Estimating the effects separately enabled us to explore the significance of the various mechanisms. Table 1 summarises the four potential mechanisms.

**Table 1:** Potential mechanisms for the impact of DTC telemedicine on subsequent healthcare consumption

Mechanism	Expected impact	Time perspective
Technology: DTC telemedicine is not sufficient for solving the problem, physical examination is necessary	More subsequent face-to-face consultations for DTC telemedicine users	Short term
Provider incentives: DTC telemedicine providers are reimbursed based on a payment per contact principle, whereas the dominating reimbursement model for primary healthcare centres is capitation. DTC telemedicine providers, therefore, have stronger financial incentives to offer additional consultations	More subsequent DTC telemedicine consultations for DTC telemedicine users	Short term
Case mix: DTC telemedicine users can be expected to be healthier than face-to-face users because of the lower thresholds for accessing healthcare	Fewer subsequent consultations (DTC telemedicine and face-to-face) for DTC telemedicine users	Short term
Patient behaviour: DTC telemedicine users adapt their healthcare-seeking behaviour and increase their use of DTC telemedicine	More subsequent DTC telemedicine consultations for DTC telemedicine users	Intermediate term

## Materials and methods

### Setting

The setting for this study was Region Stockholm, the largest region in Sweden (population 2.3 million in 2018), with the highest per capita use of DTC telemedicine in any region. In 2018, DTC telemedicine consultations accounted for 6 percent of the total number of physician consultations in primary healthcare in Region Stockholm [27].

During the study period, face-to-face and DTC telemedicine consultations differed in accessibility, patient fees and provider reimbursement. At this time, face-to-face consultations, provided by primary healthcare centres, were often preceded by a nurse triage over the phone. In Stockholm (and in Sweden), long waiting times have been one of the main issues in primary healthcare [29]. In contrast, DTC telemedicine providers have offered consultations with short waiting times [4]. To access DTC telemedicine consultations, patients download a smartphone application and use the Swedish electronic identification system (BankID) to log in. In 2018, patients in primary healthcare in Region Stockholm paid a patient fee of SEK 200 (approx. EUR 20) for a face-to-face physician consultation. For a DTC telemedicine consultation, the patient fee was slightly higher, SEK 250 (EUR 25). The difference was due to DTC telemedicine providers being contracted by another region, with fees being based on the regulations of that region. Patients aged under 18 years or over 84 years were exempt from the patient fee in primary healthcare in Region Stockholm, whereas the lower age limit for patient fees for DTC telemedicine consultations was 20 years. In terms of reimbursement, DTC telemedicine providers were reimbursed based on a payment per contact principle, whereas the dominating reimbursement for primary healthcare centres was capitation.

### Data

To quantify the amount of healthcare consumption following DTC telemedicine consultations, we constructed a database with information on healthcare consumption for all individuals residing in Region Stockholm, Sweden, on 31 December 2017 (N = 2.3 million). The database was constructed by linking national and regional registries from Statistics Sweden, the National Board of Health and Welfare, Region Jönköping (where most DTC telemedicine providers were located in 2018) and Region Stockholm. The Swedish personal identification number (unique for each individual in the Swedish population) was used for linking the different data sources. Data on DTC telemedicine consultations and face-to-face physician consultations in primary healthcare were collected from the regional healthcare registries, while data on socioeconomic variables regarding education, country of birth and income were collected from Statistics Sweden. A variable describing the number of chronic diseases was created using data from the patient registry from the National Board of Health and Welfare (for specialized healthcare) and data from the regional healthcare registries (for primary healthcare). For the variable, the number of chronic diseases registered during 2016–2017 among the 17 diseases in the Quality and Outcomes Framework [30] was calculated for each individual.

**Fig. 1:**
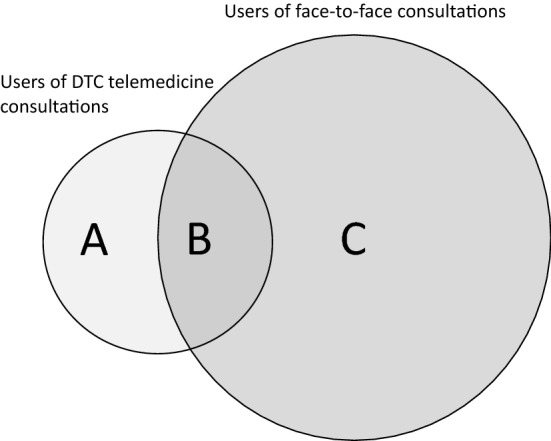
DTC telemedicine users and users of face-to-face consultation

From the constructed database, we retrieved 1,689,486 face-to-face physician consultations and 92,491 DTC telemedicine consultations conducted during the first half of 2018. One randomly selected consultation per individual was included in the analysis, implying that an individual could appear only once in the data, but that the consultation was not necessarily the individual’s first consultation during the 6-month period. The randomly selected consultations are hereafter referred to as index consultations. Several exclusions were made from the material. Individuals born in 2016 and later were excluded because their healthcare consumption could not be tracked for the full 24-month period preceding the index consultation. To ensure that index consultations initiated a care episode rather than constituting a follow-up visit, we included as index consultations only consultations that were not preceded by another consultation within a 30-day period. In addition, DTC telemedicine users were excluded from the control group. Exclusions were performed prior to randomly selecting an index consultation per individual. The last exclusion criterion meant that the control group consisted of individuals who only had face-to-face consultations and no DTC telemedicine consultations during the entire study period. In comparison, the treatment group consisted of individuals who had either only telemedicine consultations or both telemedicine consultations and face-to-face consultations during the same period. In Fig. 1, DTC telemedicine users can be found in either area A or area B, whereas controls exist only in area C. After these exclusions, the final pre-matching sample consisted of 58,978 telemedicine users and 762,106 users of face-to-face consultations.

### Matching

**Fig. 2:**
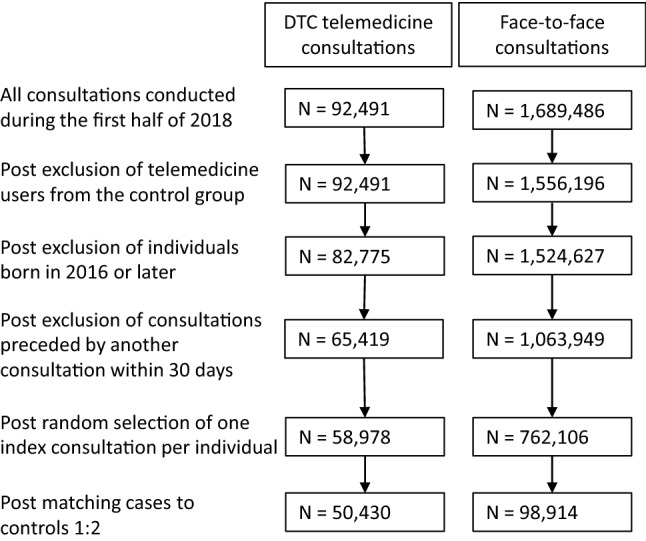
Selection of DTC telemedicine users and matched controls

Users of DTC telemedicine consultations during January–June 2018 were matched 1:2 through direct matching to a control group of non-users. The non-users had no experience of DTC telemedicine during the entire study period. However, all potential controls had a face-to-face consultation during the first half of 2018. The matching was based on a number of variables that we anticipated would impact the level and trend of healthcare utilisation (sex, age group, education, number of chronic conditions in the preceding 2-year period and diagnosis for the DTC telemedicine/face-to-face consultation at a three-digit level of the ICD-10 code, see Table 2 for classifications). After matching, the cohort of telemedicine users consisted of 50,430 individuals and the control group of 98,914 individuals (Fig. 2).

**Table 2:** Characteristics of users of DTC telemedicine and the control group of non-users before and after matching

		DTC telemedicine users	Control group	Matched DTC telemedicine users	Matched controls
		*n* = 58,978 (%)	*n* = 762,106 (%)	*n* = 50,430 (%)	*n* = 98,914 (%)
Age group (years)	0–5	14	4	13	13
	6–18	19	11	20	21
	19–25	13	6	12	12
	26–45	40	25	40	41
	46–64	12	27	13	13
	65 +	1	27	1	1
Sex	Female	59	56	58	58
	Male	41	44	42	42
Education	Lower secondary	4	14	4	4
	Upper secondary	30	37	31	31
	Post-secondary less than 3 years	20	16	19	19
	Post-secondary 3 years or more	45	31	45	45
	Missing information	1	2	1	1
ICD-10 Diagnostic chapter	A00–B99 Infectious and parasitic	11	4	8	8
	C00–D49 Neoplasms	1	1	1	1
	E00–E89 Endocrine and metabolic	1	6	1	1
	F01–F99 Mental disorders	4	5	4	4
	G00–G99 Nervous system	2	2	2	2
	H00–H59 Eye and adnexa	4	1	4	4
	H60–H95 Ear and mastoid process	1	3	2	2
	I00–I99 Circulatory system	1	10	1	1
	J00–J99 Respiratory system	23	14	26	27
	K00–K95 Digestive system	2	3	2	2
	L00–L99 Skin	13	5	13	13
	M00–M99 Musculoskeletal system	3	11	4	4
	N00–N99 Genitourinary system	6	3	5	5
	R00–R99 Symptoms unclassified elsewhere	15	16	16	17
	S00–T88 Injury and poisoning	4	2	3	3
	Z00–Z99 Factors influencing health status	7	10	7	7
	Other conditions	0	1	0	0
	Missing	1	3	1	1
Number of chronic diseases 2016–2017	0	83	60	84	85
	1	15	24	14	14
	2	2	10	2	1
	3	0	4	0	0
	4	0	1	0	0
	5 +	0	1	0	0

Before matching, DTC telemedicine users differed significantly from the control group of non-users (Table 2). DTC telemedicine users were younger than the control group and more often female. They also had higher educational levels and fewer chronic diseases. Diseases of the eye, infections and parasitic diseases and diseases of the skin were more common index consultation diagnoses among DTC telemedicine index consultations, whereas diseases of the circulatory system, endocrine and metabolic disorders and diseases of the musculoskeletal system were more common among the index consultations of the control group which consisted of face-to-face consultations. After matching, the two groups were balanced regarding these variables.

### Statistical analysis

Subsequent healthcare consumption, in terms of physician consultations following the index consultation, was analysed in a robust interrupted time series analysis in accordance with Eq. 1. Interrupted time series is a quasi-experimental design to evaluate longitudinal effects of time-delimited interventions. The design enables assessment of dynamic changes in outcomes following an intervention, while controlling for secular trends [31]. Interrupted time series analysis is especially suitable for analysing interventions that are introduced over a clearly defined time period and that target entire populations [32]. However, the approach has previously been used in examining the impact of telemedicine on healthcare utilisation [23, 24]. In these derivations of the analytical approach, the intervention analysed is use of telemedicine and the timing of the intervention is individual and vary within the study population. To analyse the effects, the intervention is anchored at time 0 and control groups are used for comparison. Having a comparison group to serve as the counterfactual is generally considered superior to single-group analysis for investigating intervention effects [33]. The underlying assumption in the analysis is that without the exposure (the intervention), the exposed group would have followed the same change in healthcare consumption as the control group.

In our analytical approach, we followed the design of McGrail and co-authors [23]. The total number of physician consultations in primary healthcare (DTC telemedicine and face-to-face) was summarized for each month of the 6-month period following the index consultation and the 24-month period preceding it. To separate the effects of face-to-face versus DTC telemedicine consultations, we used three models. In the first model, the outcome variable was the total number of physician consultations (face-to-face *and* DTC telemedicine). In the second model, the outcome variable was the number of face-to-face consultations separately and the third model included only DTC telemedicine consultations as the outcome.

1 $${Y}_{it}={\beta }_{0}+{\beta }_{1}Time+{\beta }_{2}Post\_short\_term+{\beta }_{3}Exposed+{\beta }_{4}Exposed*Time+ {\beta }_{5}Post\_short\_term*Exposed+ {\beta }_{6}Post\_intermediate\_term+ {\beta }_{7}Post\_intermediate\_term*Time+{\beta }_{8}Post\_intermediate\_term*Exposed+ {\beta }_{9}Post\_intermediate\_term{*Exposed*Time+ \varepsilon }_{it}$$

In Eq. 1, the outcome variable, ${\mathrm{Y}}_{\mathrm{it}}$, is the total number of physician consultations for individual i at period t. Time is a variable ranging from -24 to -2 and from 1 to 6, which captures the month in relation to the index consultation. We excluded observations at time -1, which was the 30-day period preceding the index consultation, and time 0, which was the day of the index consultation, since all index consultations were preceded by a washout period of 30 days with no healthcare consumption.

Post_short_term is a dummy variable taking the value 1 for observations within the first month after the index consultation (when time = 1) and 0 otherwise. Post_intermediate_term is a dummy variable taking the value 1 for the time periods 2 to 6 and 0 otherwise.

Exposed is a dummy variable taking the value 1 for the exposed individuals (users of telemedicine) and 0 for the non-exposed. Time-invariant factors that differed between the exposed group and the control group and that could influence the healthcare consumption were captured by this variable, even though we might not have observed them.

In the estimated model, ${\upbeta }_{0}$ is the intercept and ${\upbeta }_{1}$ is the difference in outcome from time t to time t + 1 before the index consultation, i.e. the pre-intervention trend, in the matched control group. ${\upbeta }_{2}$ estimates the difference in level for the control group, *in addition to the time trend,* between the level of consumption at time = − 2 and the month after the index consultation. ${\upbeta }_{3}$ estimates the difference in levels during the pre-intervention period. ${\upbeta }_{4}$ estimates the difference in pre-intervention trend between the two groups and ${\upbeta }_{5}$ estimates the difference in post-intervention levels (short term). ${\upbeta }_{6}$ estimates the change in levels after the index consultation for controls (intermediate term) and ${\upbeta }_{7}$ estimates the trend in the post-intervention period for controls. ${\upbeta }_{8}$ estimates the difference in post-intervention levels (intermediate term) and ${\upbeta }_{9}$ estimates the difference in post-intervention trends. Our main parameters of interest were ${\upbeta }_{5}$ for the short-term analysis and ${\upbeta }_{8}$ and ${\upbeta }_{9}$ for the intermediate-term analysis. Statistically significant estimates for these three parameters indicated that DTC telemedicine consultations impacted on total healthcare consumption.

The models were estimated using the statistical software SAS Enterprise Guide 7.1. A normal distribution was assumed. To deal with autocorrelation, a generalised estimating equation (GEE) model, with a repeated subject statement, was used in our estimations. The model was estimated with the proc genmod procedure in SAS Enterprise Guide 7.1. Through the repeated subject statement, the covariance structure of multivariate responses for GEE model fitting is specified, allowing for repeated measures of individuals to be correlated, while measures from different individuals are assumed to be statistically independent. To investigate the impact of seasonality, an additional model with monthly dummy variables was estimated. The adjustment for seasonality did not alter the interpretation of the results and was, therefore, not included in the final model.

## Results

The results from the interrupted time series models are displayed in Table 3. The first column shows the results from model 1, in which the outcome variable was the sum of the number of face-to-face and DTC telemedicine consultations combined. In models 2 and 3, results from model 1 are disaggregated into effects stemming from consumption of face-to-face consultations (model 2) and DTC telemedicine consultations (model 3), respectively. In model 3, controls are included in the model with outcome values of zero (by definition, controls are non-users of DTC telemedicine) to allow for a uniform model structure and presentation. Including or excluding controls from model 3 yielded the same results.

**Table 3:** Estimated values of physician healthcare consultations for users of DTC telemedicine (exposed group, *n* = 50,430) compared with matched non-users (control group, *n* = 98,914), based on an interrupted time series model

	Model 1 Y = face-to-face consultations + DTC telemedicine consultations		Model 2 Y = face-to-face consultations		Model 3 Y = DTC telemedicine consultations	
	Estimate	*P* value	Estimate	*P* value	Estimate	*P* value
β0 Intercept	0.137	< 0.001	0.137	< 0.001		
β1 Existing trend for controls	< 0.001	< 0.001	< 0.001	< 0.001		
β2 Change in level after index consultation for controls (short term)	0.081	< 0.001	0.081	< 0.001		
β3 Difference in level in pre-intervention period	0.013	< 0.001	− 0.035	< 0.001	0.048	< 0.001
β4 Difference in existing trend	0.001	< 0.001	− 0.001	< 0.001	0.002	< 0.001
β5 Difference in post-intervention level (short term)	0.087	< 0.001	0.025	< 0.001	0.062	< 0.001
β6 Change in level after index consultation for controls (intermediate term)	− 0.003	0.081	− 0.003	0.081		
β7 Trend in post-intervention period for controls	− 0.002	< 0.001	− 0.002	< 0.001		
β8 Difference in post-intervention level (intermediate term)	0.017	< 0.001	0.012	< 0.001	0.005	0.001
β9 Difference in post-intervention trend	− 0.002	0.009	< 0.001	0.930	− 0.002	< 0.001

### Level of pre-intervention consumption

According to model 1, DTC telemedicine users had an increasing trend in their consumption of physician consultations in the pre-intervention period (${\upbeta }_{4}$, model 1), which was entirely attributed to an increasing use of DTC telemedicine (${\upbeta }_{4}$, model 3), while the trend for face-to-face consultations was decreasing (${\upbeta }_{4}$, model 2). The level of consumption shortly before the index consultation was higher for DTC telemedicine users than for the control group (${\upbeta }_{3}$, model 1). Again, this result was driven by consumption of DTC telemedicine consultations, since their pre-intervention level of face-to-face consultations was lower than that for the control group (${\upbeta }_{3}$, model 2).

### Short-term effects

Regarding the short-term effects, our main parameter of interest was ${\upbeta }_{5}$, which captured the change in levels of healthcare consumption (in addition to the pre-intervention trend), from before the index consultation (t = − 2) to the first observation after the index consultation (t = 1). This level was significantly higher for the DTC telemedicine users than for controls. The change in levels was 0.087 consultations in total between the groups (${\upbeta }_{5}$, model 1). Approximately one third of the change in levels could be attributed to face-to-face consultations (0.025) and according to model 3, approximately two thirds could be attributed to DTC telemedicine consultations (0.062).

### Intermediate-term effects

Regarding intermediate-term effects, the main parameters of interest were ${\upbeta }_{8}$ and ${\upbeta }_{9}$. ${\upbeta }_{8}$ estimated the change in levels of healthcare consumption, in addition to the pre-intervention trend, from before the index consultation (at t = − 2) to the second observation after the index consultation (t = 2). The positive value of ${\upbeta }_{8}$ in model 1 indicated that DTC telemedicine consultations increased primary healthcare utilisation in the intermediate term as well. However, the intermediate-term effect was much more modest than the short-term effect (0.017 vs. 0.087). Regarding months 2–6, the negative value of the interaction term ${\upbeta }_{9}$ indicated that the difference between DTC telemedicine users and matched controls decreased over time.

### Total effect

If we assume that the DTC telemedicine users would have followed the same changes in trend and in level as the control group in the absence of DTC telemedicine, we can estimate the counterfactual level of consumption for the entire follow-up period. The counterfactual level of consumption for the exposed group summarized for the short-term and intermediate-term periods (excluding the day of the index consultation) would have been 0.962 physician consultation for these 6 months. The predicted level of consultations according to the model for the summarized time period was 1.097 physician consultations. Consequently, the increase in the actual scenario in comparison to the counterfactual scenario was 0.135 consultations, which represents an increase of 14 percent. For the total sample, the additional consumption for DTC telemedicine users over the entire 6-month follow-up period was attributed more to the short-term effect (0.087 additional consultations) than the intermediate-term effect (0.048 additional consultations).

### Graphical illustration

**Fig. 3:**
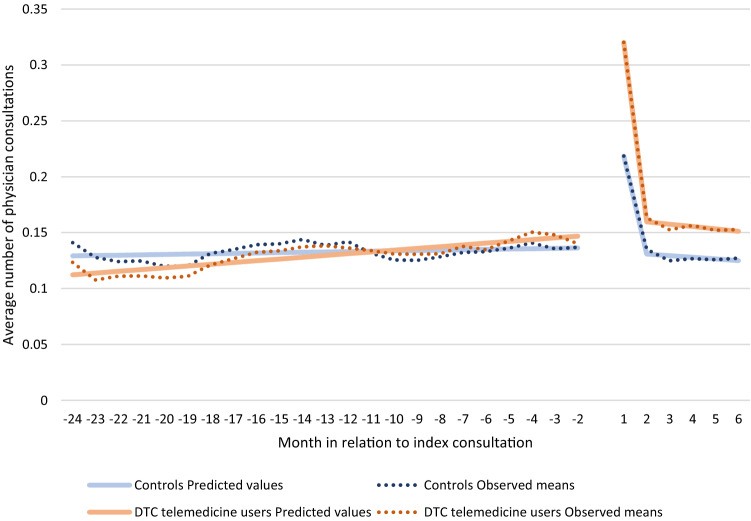
Observed and predicted numbers of total physician consultations per month in the 24-month period preceding and the 6-month period following an index consultation for DTC telemedicine users and a matched control group of non-users

Figure 3 illustrates the results from the estimations and the observed means for the two groups. The observed mean values for each group and month are presented as dotted lines, whereas the predicted values from the interrupted time series model are displayed as solid lines. Both the predicted values and the observed means show that DTC telemedicine users had a significantly higher increase in their healthcare consumption in the short term and a slightly higher increase in the intermediate term, compared with the control group.

### Estimations by age group and diagnosis

**Fig. 4:**
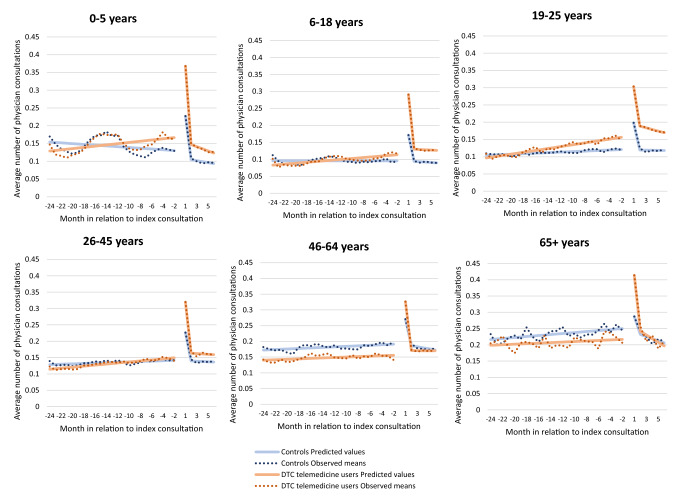
Observed and predicted numbers of total physician consultations per month in the 24-month period preceding and the 6-month period following an index consultation for DTC telemedicine users and a matched control group of non-users, by age group

To investigate the robustness of the results, we stratified the analysis by age group and by diagnostic chapter. Table 4 and Fig. 4 show the results of the age-stratified analyses. For two of our main parameters of interest, i.e. the difference in post-intervention levels, in the short (${\upbeta }_{5}$) and intermediate term (${\upbeta }_{8}$), the estimated values were positive across all age groups in model 1, indicating a larger increase in primary healthcare consumption for DTC telemedicine users than for controls. However, the differences in post-intervention trends (${\upbeta }_{9})$ were statistically significant only for the two age groups 0–5 years and 19–25 years. For these age groups, the estimated values were negative, in accordance with the main model in Table 3.

**Table 4:** Estimated values of physician healthcare consultations for users of DTC telemedicine (exposed group) in comparison to non-users (control group), stratified by age group, based on an interrupted time series model

		0–5 *n* = 18,768		6–18 *n* = 30,687		19–25 *n* = 17,510		26–45 *n* = 60,491		46–64 *n* = 19,705		65 + *n* = 2183	
		Estimate	*P* value	Estimate	*P* value	Estimate	*P* value	Estimate	*P* value	Estimate	*P* value	Estimate	*P* value
Model 1	β0 Intercept	0.128	< 0.001	0.096	< 0.001	0.122	< 0.001	0.143	< 0.001	0.193	< 0.001	0.253	< 0.001
	β1 Existing trend for controls	− 0.001	< 0.001	< 0.001	0.878	0.001	< 0.001	0.001	< 0.001	0.001	< 0.001	0.002	0.001
	β2 Change in level after index consultation for controls (short term)	0.100	< 0.001	0.076	< 0.001	0.075	< 0.001	0.083	< 0.001	0.077	< 0.001	0.033	0.049
	β3 Difference in level in pre-intervention period	0.042	< 0.001	0.021	< 0.001	0.039	< 0.001	0.010	< 0.001	− 0.035	< 0.001	− 0.035	0.009
	β4 Difference in existing trend	0.003	< 0.001	0.001	< 0.001	0.002	< 0.001	0.001	< 0.001	< 0.001	0.550	− 0.001	0.359
	β5 Difference in post-intervention level (short term)	0.096	< 0.001	0.097	< 0.001	0.064	< 0.001	0.083	< 0.001	0.091	< 0.001	0.162	< 0.001
	β6 Change in level after index consultation for controls (intermediate term)	− 0.018	< 0.001	0.001	0.815	− 0.002	0.633	− 0.001	0.625	< 0.001	0.982	− 0.008	0.663
	β7 Trend in post-intervention period for controls	− 0.002	0.090	− 0.001	0.129	− 0.001	0.327	− 0.001	0.027	− 0.004	0.001	− 0.008	0.044
	β8 Difference in post-intervention level (intermediate term)	0.007	0.436	0.015	0.027	0.039	< 0.001	0.015	0.005	0.014	0.157	0.054	0.099
	β9 Difference in post-intervention trend	− 0.006	0.002	− 0.001	0.418	− 0.006	0.004	− 0.001	0.263	0.003	0.130	− 0.004	0.559
Model 2	β0 Intercept	0.128	< 0.001	0.096	< 0.001	0.122	< 0.001	0.143	< 0.001	0.193	< 0.001	0.253	< 0.001
	β1 Existing trend for controls	− 0.001	< 0.001	< 0.001	0.878	0.001	< 0.001	0.001	< 0.001	0.001	< 0.001	0.002	0.001
	β2 Change in level after index consultation for controls (short term)	0.100	< 0.001	0.076	< 0.001	0.075	< 0.001	0.083	< 0.001	0.077	< 0.001	0.033	0.049
	β3 Difference in level in pre-intervention period	− 0.028	< 0.001	− 0.020	< 0.001	− 0.015	< 0.001	− 0.038	< 0.001	− 0.072	< 0.001	− 0.066	< 0.001
	β4 Difference in existing trend	< 0.001	0.254	− 0.001	< 0.001	− 0.001	0.021	− 0.001	< 0.001	− 0.002	< 0.001	− 0.002	0.007
	β5 Difference in post-intervention level (short term)	0.008	0.322	0.024	< 0.001	− 0.008	0.332	0.030	< 0.001	0.046	< 0.001	0.115	< 0.001
	β6 Change in level after index consultation for controls (intermediate term)	− 0.018	< 0.001	0.001	0.815	− 0.002	0.633	− 0.001	0.625	< 0.001	0.982	− 0.008	0.663
	β7 Trend in post-intervention period for controls	− 0.002	0.090	− 0.001	0.129	− 0.001	0.327	− 0.001	0.027	− 0.004	0.001	− 0.008	0.044
	β8 Difference in post-intervention level (intermediate term)	0.016	0.027	− 0.001	0.860	0.015	0.080	0.014	0.003	0.016	0.074	0.052	0.092
	β9 Difference in post-intervention trend	− 0.003	0.072	0.001	0.662	− 0.003	0.144	< 0.001	0.867	0.004	0.076	− 0.002	0.823
Model 3	β0 Intercept												
	β1 Existing trend for controls												
	β2 Change in level after index consultation for controls (short term)												
	β3 Difference in level in pre-intervention period	0.070	< 0.001	0.041	< 0.001	0.054	< 0.001	0.048	< 0.001	0.036	< 0.001	0.032	< 0.001
	β4 Difference in existing trend	0.003	< 0.001	0.002	< 0.001	0.002	< 0.001	0.002	< 0.001	0.002	< 0.001	0.001	< 0.001
	β5 Difference in post-intervention level (short term)	0.088	< 0.001	0.073	< 0.001	0.071	< 0.001	0.053	< 0.001	0.045	< 0.001	0.047	< 0.001
	β6 Change in level after index consultation for controls (intermediate term)												
	β7 Trend in post-intervention period for controls												
	β8 Difference in post-intervention level (intermediate term)	− 0.009	0.066	0.016	< 0.001	0.024	< 0.001	0.001	0.640	− 0.002	0.503	0.002	0.858
	β9 Difference in post-intervention trend	− 0.003	0.003	− 0.002	0.043	− 0.004	0.002	− 0.002	0.005	< 0.001	0.739	− 0.003	0.256

In model 1, the outcome variable is the sum of the number of face-to-face and *DTC* telemedicine consultations combined. In model 2, the outcome variable is based on face-to-face consultations only, and in model 3 on *DTC* telemedicine consultations only

Younger age was associated with a larger increase in DTC telemedicine consultations in the short term (${\upbeta }_{5}$, model 3). Regarding the intermediate-term increase, ${\upbeta }_{8}$ from models 2 and 3 showed that the increase was mostly determined by DTC telemedicine consultations for the age groups 6–18 years and 19–25 years.

In addition, the age-stratified analysis revealed that the differences in levels during the pre-intervention period varied across age groups. The increase in physician consultations during the pre-intervention period (${\upbeta }_{4}$, model 1) was greater in the age groups 0–5 years and 19–25 years than in other age groups and was driven by an increasing use of DTC telemedicine consultations (${\upbeta }_{4}$, model 2 and 3). In the oldest age groups, the level of consumption was lower in the pre-intervention period for DTC telemedicine users than for the control group (${\upbeta }_{3}$, model 1) Table 4.

Table 5 displays the results of model 1 stratified by diagnostic chapter. The results are based on the six most common diagnostic chapters among DTC telemedicine consultations and cover 76 percent of the index consultations included in the study. As shown in Table 5, the main results are robust across diagnostic chapters. DTC telemedicine consultations are associated with a larger subsequent healthcare consumption, especially in the short term. However, there are some variations across the chapters. For *Diseases of the genitourinary system,* the differences in short-term and intermediate-term consumption subsequent to DTC telemedicine and face-to-face consultations are not statistically significant. In contrast, the intermediate-term additional consumption for *Diseases of the skin* was particularly pronounced.

**Table 5:** Estimated values of physician healthcare consultations for users of DTC telemedicine (exposed group) in comparison to non-users (control group), stratified by diagnostic chapter of index consultation, based on an interrupted time series mode

	J00–J99 Diseases of the respiratory system *n* = 39,769		R00–R99 Symptoms not elsewhere classified *n* = 24,692		L00–L99 Diseases of the skin *n* = 19,216		A00–B99 Infectious and parasitic diseases *n* = 12,221		Z00–Z99 Factors influencing health status *n* = 10,376		N00–N99 Diseases of the genitourinary system *n* = 8117	
	Estimate	*P* value	Estimate	*P* value	Estimate	*P* value	Estimate	*P* value	Estimate	*P* value	Estimate	*P* value
β0 Intercept	0.131	< 0.001	0.137	< 0.001	0.111	< 0.001	0.112	< 0.001	0.135	< 0.001	0.167	< 0.001
β1 Existing trend for controls	< 0.001	0.088	0.001	< 0.001	< 0.001	0.051	< 0.001	0.043	< 0.001	0.574	< 0.001	0.039
β2 Change in level after index consultation for controls (short term)	0.098	< 0.001	0.088	< 0.001	0.075	< 0.001	0.082	< 0.001	0.050	< 0.001	0.094	< 0.001
β3 Difference in level in pre-intervention period	− 0.001	0.756	0.014	< 0.001	0.008	0.013	0.008	0.047	0.053	< 0.001	− 0.003	0.661
β4 Difference in existing trend	0.001	< 0.001	0.001	< 0.001	0.001	< 0.001	0.001	< 0.001	0.003	< 0.001	0.001	0.047
β5 Difference in post-intervention level (short term)	0.106	< 0.001	0.109	< 0.001	0.077	< 0.001	0.070	< 0.001	0.065	< 0.001	0.016	0.246
β6 Change in level after index consultation for controls (intermediate term)	− 0.009	0.006	0.007	0.134	− 0.003	0.552	− 0.005	0.357	0.015	0.038	− 0.020	0.023
β7 Trend in post-intervention period for controls	0.001	0.409	− 0.004	< 0.001	− 0.002	0.045	< 0.001	0.745	− 0.003	0.028	0.002	0.218
β8 Difference in post-intervention level (intermediate term)	0.018	0.004	0.013	0.120	0.047	< 0.001	0.017	0.104	− 0.019	0.164	0.011	0.478
β9 Difference in post-intervention trend	− 0.002	0.128	− 0.001	0.447	− 0.003	0.170	− 0.001	0.630	− 0.002	0.491	0.002	0.571

## Discussion

The aim of this study was to assess the impact of telemedicine consultations on subsequent consumption of physician consultations in primary healthcare. The results indicated that DTC telemedicine consultations increased the total number of physician consultations in primary healthcare. However, the increased consumption seemed to be short term rather than intermediate term. The results were robust across all investigated age groups, except the differences in post-intervention trends, which were not significant for all age groups. There were some variations across diagnostic chapters, indicating that DTC telemedicine substituted face-to-face consultations to a greater extent for diseases of the genitourinary system (such as urinary tract infections) whereas for diseases of the skin, DTC telemedicine met previously unmet demands for healthcare to a greater extent.

### Technology

The results from model 2 indicated that the DTC telemedicine users increased their levels of face-to-face consultations after the index consultation to a greater extent than the control group. This result gives support for the first potential mechanism presented in Table 1. DTC telemedicine consultations may be less efficient than face-to-face consultations in solving acute health problems. However, it could also be the case that DTC telemedicine is more appropriate for dealing with certain types of diseases, while other types require the wider range of diagnostic tools offered in face-to-face consultations. The analysis stratified by diagnostic chapter indicated that DTC telemedicine consultations had differing potential in different disease areas.

### Provider incentives

The high level of DTC telemedicine consultations in the short-term follow-up period also gave some support for the second mechanism presented in Table 1. DTC telemedicine providers may initiate follow-up contacts to a greater extent than providers of face-to-face consultations. Two thirds of the additional consumption in the short term could be attributed to DTC telemedicine consultations and one third to face-to-face consultations. DTC telemedicine providers were reimbursed based on a payment per contact principle during the study period and, therefore, had strong incentives for increasing visit volumes, possibly at the risk of providing unnecessary consultations [25]. In contrast, primary healthcare centres are reimbursed mainly through capitation, which leads to lower incentives for providing appointments and follow-ups.

According to the Swedish Competition Authority, the differing compensation mechanisms for DTC telemedicine providers and traditional primary healthcare providers restrict competition on the primary healthcare market. The reimbursement for integrated telemedicine consultations offered by traditional primary healthcare facilities is less than that for DTC telemedicine consultations, making it difficult for primary healthcare centres to offer comparable telemedicine services and remain competitive [34].

### Patient case mix

Another potential mechanism in the short-term perspective, working in the opposite direction to the mechanisms mentioned above, is the potential difference in case mix between users of DTC telemedicine and users of face-to-face consultations. Since DTC telemedicine has a lower barrier to care than that seen in traditional primary healthcare settings, there is a possibility that the medical conditions of DTC telemedicine users are less severe, on average, than those of face-to-face users. We matched DTC telemedicine users with controls based on several well-known influencing factors, including visit diagnosis, to reduce any differences in healthcare consumption due to underlying individual traits. However, selection bias might still have led us to underestimate additional consumption among DTC telemedicine users [35].

### Patient behaviour

For the intermediate-term impact, we mainly explored if DTC telemedicine consultations changed subsequent health-seeking behaviour. The intermediate-term effect was not as pronounced as the short-term effect. Still, DTC telemedicine users had a higher increase of consumption than controls also in the intermediate term, in comparison to pre-intervention levels. A potential explanation could be that healthcare-seeking behaviour changes after a DTC telemedicine consultation; a positive experience might lead to additional consumption. However, the effect was mainly explained by additional face-to-face consultations, not by DTC telemedicine consultations. This effect may be caused by the technological limitations of DTC telemedicine with respect to physical examinations and information exchange. Thus, the pattern we observed may be related to the first mechanism presented in Table 1, spilling over from the short term to the intermediate term.

### Comparison to previous studies

Our results were consistent with studies suggesting that DTC telemedicine visits increase short-term follow-up visits within 1 month from the initial visit, taking account of follow-up for the same condition and other conditions [5, 9, 10, 14, 36]. The summarized effect of the entire follow-up period led to an increase in the number of physician consultations for the exposed group in comparison to the counterfactual scenario. These results were consistent with studies suggesting that DTC telemedicine increases overall healthcare consumption [5, 18–22]. However, these findings contrast to those of McGrail and co-authors [23], whose study design we followed and who found that telemedicine users had lower expenditure than their matched controls at the end of the follow-up period. The results were the same irrespective of whether the outcome was measured in number of consultations or costs. There are several factors that may explain this difference in results. McGrail and co-authors investigated another type of healthcare service that seem to be more integrated in the traditional provision of healthcare, whereas in our setting, the DTC telemedicine providers operated in parallel to traditional primary healthcare providers. Integration in regular healthcare services might enable more efficient use of resources. Another difference in approach is that we use the index consultation diagnosis as a matching variable. The differences in diagnoses for telemedicine users and controls may have affected their subsequent healthcare consumption to varying extent. A third difference was the difference in follow-up period. Our data were restricted to a follow-up period of 6 months whereas McGrail and co-authors followed their patients for 18 months.

### Policy implications

Shaping the future role of telemedicine is high on the agenda in many healthcare systems as a consequence of the increased use of telemedicine during the COVID-19 pandemic [37]. This study sheds light on the early experiences of telemedicine in a pre-pandemic setting, providing lessons that might be applicable in a post-pandemic setting as well.

The results indicate that DTC telemedicine increases the overall consumption in primary healthcare. The effect is especially prevalent in the short term after an index consultation, which indicates that DTC telemedicine consultations are less efficient than face-to-face consultations in solving the acute health problem. However, this does not automatically imply that medical spending will increase with increased use of DTC telemedicine. To evaluate the economic consequences of telemedicine visits, it is necessary to consider both the cost differences between the two consultation types, and how telemedicine consultations affect overall healthcare consumption. Previous research suggests that the cost per episode is lower for telemedicine users than for face-to-face users [3, 5, 15, 17]. However, the few studies that have examined how telemedicine visits affect overall spending are ambiguous. Some studies indicate that the cost savings related to less expensive care episodes might be outweighed by the increased cost due to new utilisation [5, 38], whereas others indicate that telemedicine consultations are likely to decrease overall medical spending [15, 23, 39].

Irrespective of whether or not DTC telemedicine mitigates rising costs, it can be beneficial from other perspectives. Previous research suggests that telemedicine consultations are appreciated by patients [6, 40–43]. Moreover, the overall impact of telemedicine consultations on the healthcare system depends on the extent to which the historical capacity was sufficient to meet the patients’ care needs. In general, limited accessibility is considered a major issue in Swedish primary healthcare. In comparison to similar healthcare systems, the resources allocated to the primary healthcare sector, e.g. general practitioners per capita, are small [44]. In contrast, DTC telemedicine providers have offered almost immediate access to qualified healthcare assessments, which has been perceived as one of the most positive aspects of their services [4].

The increased healthcare consumption for DTC telemedicine users, attributed to increased use of DTC telemedicine (model 3), was more pronounced in the younger age groups, which indicates that younger individuals are more likely to adapt to telemedicine as an alternative to traditional healthcare services. This finding is also confirmed by Table 2, which shows that DTC telemedicine users were much younger than face-to-face users before matching. An expansion of telemedicine could consequently lead to a reallocation of healthcare resources from older to younger age groups.

In terms of generalizability, our results were based on a setting where DTC telemedicine providers operated in parallel with the regular primary healthcare system. If we had examined telemedicine consultations offered as an integrated part of the care provided by primary healthcare centres also offering face-to-face visits, the results might have been different. For example, McGrail and co-authors [23] suggested that continuity, defined as getting to consult with a known provider digitally, substantially decreased both costs and healthcare consumption. Moreover, our study was limited to the Swedish context, in which healthcare is publicly financed with universal coverage. Therefore, our findings may not be generalizable to healthcare systems based on private insurance or where patients are faced with the full costs of telemedicine contacts. However, from the patient perspective, the effect of DTC telemedicine on subsequent healthcare consumption is likely to be similar, as long as a third-party payer is involved [22].

### Limitations

The main limitation of the study is the risk of selection bias in the two compared groups. The longitudinal data allowed us to control for time-invariant unobservable and observable patient characteristics that could influence healthcare consumption. However, we could not control for unobservable time-varying patient characteristics that influenced healthcare consumption.

We recognise that we would have had a stronger case to establish a causal claim if we had been able to compare a context in which telemedicine was implemented on a wide scale in a short period of time within a setting where it had not previously been implemented. Due to the gradual growth of telemedicine in Sweden, this was not possible. Even though our use of the interrupted time series analysis with different intervention periods deviated from its original usage, the fact that DTC users and controls showed similar pre-intervention consumption patterns provided credibility to our identification strategy and gave support for a causal effect of DTC telemedicine on subsequent primary healthcare consultations.

An additional limitation of the analysis is that it was restricted to physician consultations in primary healthcare. Expanding the outcome measure to additional healthcare professionals and other sectors of the healthcare system might have generated other results. Another limitation concerns the fact that we did not separate the effects for first-time users from those for recurrent users. In future studies, it would be interesting to analyse these two groups separately.

## Conclusion

The aim of this study was to assess the short- and intermediate-term impact of DTC telemedicine consultations on overall utilisation of primary care, by comparing users of DTC telemedicine to matched controls in a Swedish setting. The results indicated that telemedicine consultations increased the total number of physician consultations in primary healthcare. We observed short-term effects for DTC telemedicine users, implying more face-to-face and remote follow-ups. The effects can be connected to both technological limitations and provider incentives; the first and second mechanisms of our conceptual framework to categorise the impact of DTC telemedicine on healthcare consumption. From a policy perspective, it is therefore important to further investigate for which diagnoses and treatments DTC telemedicine is suitable, so that its use can be encouraged when it is most cost-efficient and limited when it is not. Given the fundamentally different reimbursement models, there are reasons to review and possibly harmonize the incentive structures for both types of services. The third mechanism, relating to case mix differences, probably impacted on our results to a lesser degree because of the approach of matching telemedicine users to controls using face-to-face consultations.

The additional consumption for DTC telemedicine users was especially pronounced in the short-term perspective but was also present in the intermediate term. The results indicated that DTC telemedicine users adapted their behaviour to the new landscape of primary care, with DTC telemedicine providers offering more convenient and accessible healthcare services, giving support to the fourth mechanism. We identified several possible drivers behind the increased consumption of DTC telemedicine. There may be a previously unmet demand for healthcare that gained an outlet. The increase may also have related to limitations of technology and patients’ need for information. Further research is needed to properly investigate and understand the underlying causes for the fourth mechanism.

The increased consumption is not necessarily synonymous with increased expenditure but the age profile of the users indicated that expansion of the services could lead to a reallocation of healthcare resources from older to younger age groups.

## References

[1] Ekman B (2018). Cost Analysis of a digital health care model in Sweden. PharmacoEco Open..

[2] Dullet NW, Geraghty EM, Kaufman T, Kissee JL, King J, Dharmar M (2017). Impact of a university-based outpatient telemedicine program on time savings, travel costs, and environmental pollutants Value in Health. J Inter Soc Pharma Out Res.

[3] Gordon AS, Adamson WC, DeVries AR (2017). Virtual visits for acute, nonurgent care: a claims analysis of episode-level utilization. J Med Int Res..

[4] Gabrielsson-Järhult F, Kjellström S, Josefsson KA (2021). Telemedicine consultations with physicians in Swedish primary care: a mixed methods study of users' experiences and care patterns. Scandinavian J Primary Health Care..

[5] Ashwood JS, Mehrotra A, Cowling D, Uscher-Pines L (2017). &nbsp;Direct-to-consumer telehealth may increase access to care but does not decrease spending. Health Aff..

[6] Farr M, Banks J, Edwards HB, Northstone K, Bernard E, Salisbury C (2018). Implementing online consultations in primary care: a mixed-method evaluation extending normalisation process theory through service co-production. BMJ Open.

[7] Blix M, Levay C (2018). Digitalization and Health Care. Eso expertgrupp.

[8] Johnson KM, Dumkow LE, Burns KW, Yee MA, Egwuatu NE (2019). Comparison of diagnosis and prescribing practices between virtual visits and office visits for adults diagnosed with sinusitis within a primary care network. Open forum infect dis.

[9] Penza KS, Murray MA, Myers JF, Maxson J, Furst JW, Pecina  JL (2020). Treating pediatric conjunctivitis without an exam: An evaluation of outcomes and antibiotic usage. J. Telemed. Telecare.

[10] Shi Z, Mehrotra A, Gidengil CA, Poon SJ, Uscher-Pines L, Ray KN (2018). Quality of care for acute respiratory infections during direct-to-consumer telemedicine visits for adults. Health Aff..

[11] Murray MA, Penza KS, Myers JF, Furst JW, Pecina JL (2020). Comparison of eVisit management of urinary symptoms and urinary tract infections with standard care. Telemed. J. E Health.

[12] Tan LF, Mason N, Gonzaga WJ (2017). Virtual visits for upper respiratory tract infections in adults associated with positive outcome in a Cox model. Telemed. J. E Health.

[13] Mehrotra A, Paone S, Martich GD, Albert SM, Shevchik GJ (2013). &nbsp;A comparison of care at e-visits and physician office visits for sinusitis and urinary tract infection. JAMA Intern. Med..

[14] Hertzog R, Johnson J, Smith J, McStay FW, da Graca B, Haneke T (2019). Diagnostic accuracy in primary care e-visits: Evaluation of a large integrated health care delivery system's experience. Mayo Clin. Proc..

[15] Lovell T, Albritton J, Dalto J, Ledward C, Daines W (2021). Virtual vs traditional care settings for low-acuity urgent conditions: An economic analysis of cost and utilization using claims data. J. Telemed. Telecare.

[16] Uscher-Pines L, Mehrotra A (2014). Analysis of Teladoc use seems to indicate expanded access to care for patients without prior connection to a provider. Health Aff..

[17] Courneya PT, Palattao KJ, Gallagher JM (2013). HealthPartners' online clinic for simple conditions delivers savings of $88 per episode and high patient approval. Health Aff..

[18] Bavafa H, Hitt LM, Terwiesch C (2018). Terwiesch C: The impact of e-visits on visit frequencies and patient health: Evidence from primary care. Manage. Sci..

[19] Ellegård L., Kjellsson, G.: Nätvårdsanvändare i Skåne kontaktade oftare vårdcentral. Läkartidningen.116:FSWP (2019). 31638708

[20] Pearl R (2014). Kaiser Permanente Northern California: current experiences with internet, mobile, and video technologies. Health Aff..

[21] North F, Crane SJ, Chaudhry R, Ebbert JO, Ytterberg K, Tulledge-Scheitel SM (2014). Impact of patient portal secure messages and electronic visits on adult primary care office visits. Telemed. J. E Health.

[22] Ellegård, L. M., Kjellsson, G., Mattisson, L.: An App Call a Day Keeps the Patient Away? Substitution of Online and In-Person Doctor Consultations Among Young Adults. Working Paper in Economics, Department of Economics, University of Gothenburg, Göteborg (2021)

[23] McGrail KM, Ahuja MA, Leaver CA (2017). Virtual visits and patient-centered care: Results of a patient survey and observational study. J Med Int Res..

[24] Shah SJ, Schwamm LH, Cohen AB, Simoni MR, Estrada J, Matiello M (2018). Virtual visits partially replaced in-person visits in an aco-based medical specialty practice. Health Aff..

[25] Jegers M, Kesteloot K, De Graeve D, Gilles W (2002). A typology for provider payment systems in health care. Health Policy.

[26] Lindgren P.: Ersättningen och e-hälsan. SNS Förlag (2019)

[27] Dahlgren C, Dackehag M, Wändell P, Rehnberg  C (2021). Determinants for use of direct-to-consumer telemedicine consultations in primary healthcare-a registry based total population study from Stockholm, Sweden. BMC Fam Pract..

[28] van Loenen T, van den Berg MJ, Faber MJ, Westert GP (2015). Propensity to seek healthcare in different healthcare systems: analysis of patient data in 34 countries. BMC Health Ser Res..

[29] Vårdanalys.: Vården ur befolkningens perspektiv–en jämförelse mellan Sverige och tio andra länder [the Commonwealth Fund’s 2016 international health policy survey of adults in 11 countries (2016)

[30] Brilleman SL, Gravelle H, Hollinghurst S, Purdy S, Salisbury C, Windmeijer F (2014). Keep it simple? Predicting primary health care costs with clinical morbidity measures. J. Health Econ..

[31] Wagner AK, Soumerai SB, Zhang F, Ross-Degnan D (2002). Segmented regression analysis of interrupted time series studies in medication use research. J. Clin. Pharm. Ther..

[32] Bernal JL, Cummins S, Gasparrini A (2017). Interrupted time series regression for the evaluation of public health interventions: A tutorial. Int. J. Epidemiol..

[33] Linden A (2017). Challenges to validity in single-group interrupted time series analysis. J. Eval. Clin. Pract..

[34] Konkurrensverket: Privata digitala vårdtjänsters påverkan på konkurrensförhållanden inom primärvården. 2022:3 (2022)

[35] Zhao M, Hamadi H, Haley DR, Xu J, White-Williams C, Park S (2020). Telehealth: advances in alternative payment models. Telemed Health..

[36] Johnson KM, Dumkow LE, Burns KW, Yee MA, Egwuatu NE (2019). Comparison of diagnosis and prescribing practices between virtual visits and office visits for adults diagnosed with sinusitis within a primary care network. Open Forum Infect Dis..

[37] Garattini L, Badinella MM, Zanetti M (2021). More room for telemedicine after COVID-19: lessons for primary care?. Springer..

[38] Newbould J, Abel G, Ball S, Corbett J, Elliott M, Exley J (2017). Evaluation of telephone first approach to demand management in English general practice: Observational study. BMJ.

[39] Nord G, Rising KL, Band RA, Carr BG, Hollander JE (2019). On-demand synchronous audio video telemedicine visits are cost effective. Am. J. Emerg. Med..

[40] Carter M, Fletcher E, Sansom A, Warren FC, Campbell JL (2018). Feasibility, acceptability and effectiveness of an online alternative to face-to-face consultation in general practice: A mixed-methods study of webGP in six Devon practices. BMJ Open.

[41] Martinez KA, Rood M, Jhangiani N, Kou L, Rose S, Boissy A (2018). Patterns of use and correlates of patient satisfaction with a large nationwide direct to consumer telemedicine service. J. Gen. Intern. Med..

[42] Foster CB, Martinez KA, Sabella C, Weaver GP, Rothberg MB (2019). Patient satisfaction and antibiotic prescribing for respiratory infections by telemedicine. Pediatrics.

[43] Powell RE, Stone D, Hollander JE (2018). Patient and health system experience with implementation of an enterprise-wide telehealth scheduled video visit program: Mixed-methods study. JMIR Med Inform..

[44] Anell A (2015). The public-private pendulum–patient choice and equity in Sweden. N Engl J Med..

